# Rare high-impact disease variants: properties and identifications

**DOI:** 10.1017/S0016672316000033

**Published:** 2016-03-21

**Authors:** LEEYOUNG PARK, JU HAN KIM

**Affiliations:** 1 Natural Science Research Institute, Yonsei University, 134 Shinchon-Dong, Seodaemun-Gu, Seoul, 120-749, Korea; 2 Seoul National University Biomedical Informatics (SNUBI), Seoul National University College of Medicine, Seoul 110-799, Korea; 3 Systems Biomedical Informatics National Core Research Center (SBI-NCRC), Seoul National University College of Medicine, 103 Daehak-ro, Jongno-gu, Seoul, 110-799, Korea

## Abstract

Although many genome-wide association studies have been performed, the identification of disease polymorphisms remains important. It is now suspected that many rare disease variants induce the association signal of common variants in linkage disequilibrium (LD). Based on recent development of genetic models, the current study provides explanations of the existence of rare variants with high impacts and common variants with low impacts. Disease variants are neither necessary nor sufficient due to gene–gene or gene–environment interactions. A new method was developed based on theoretical aspects to identify both rare and common disease variants by their genotypes. Common disease variants were identified with relatively small odds ratios and relatively small sample sizes, except for specific situations in which the disease variants were in strong LD with a variant with a higher frequency. Rare disease variants with small impacts were difficult to identify without increasing sample sizes; however, the method was reasonably accurate for rare disease variants with high impacts. For rare variants, dominant variants generally showed better Type II error rates than recessive variants; however, the trend was reversed for common variants. Type II error rates increased in gene regions containing more than two disease variants because the more common variant, rather than both disease variants, was usually identified. The proposed method would be useful for identifying common disease variants with small impacts and rare disease variants with large impacts when disease variants have the same effects on disease presentation.

## 1. Introduction

Genome-wide association studies (GWAS) have been successful in revealing the existence of common disease variants; however, common variants identified using GWAS explain only small portions of heritability (Manolio *et al.*, 2009). This prompted efforts to find rare disease variants using re-sequencing to explain the remaining causes of heritability (Cirulli & Goldstein, 2010). In addition to finding rare disease variants, it was suggested that GWAS signals could be synthetic due to rare disease variants (Dickson *et al.*, 2010). Recent studies provided evidence of the synthetic associations of common variants due to rare disease variants (Fellay *et al.*, 2010; Saunders *et al.*, 2014). However, studies have also indicated that common variants with small effects are mainly responsible for complex traits (Morrison *et al.*, 2013; Gaugler *et al.*, 2014). It is clear that both common and rare variants are responsible for disease presentation, and studies should focus on how these combined effects explain how variants cause disease (Gibson, 2011).

When integrating the effects of variants, it is most efficient to apply the effects of actual disease variants rather than those of variants with indirect associations; however, the functions of variants are difficult to predict in most cases (Cordell & Clayton, 2005). The first report to identify actual functional variants through GWAS was for differential drug responses in patients with chronic hepatitis C (Fellay *et al.*, 2010). Two *ITPA* gene variants with known functions were found. Using a regressive model, two functional variants (A/a and B/b) entirely explained the GWAS signal (C/c). For both functional variants, each minor allele was linked to the major allele of another functional variant, and both minor alleles were strongly linked to the minor alleles of the GWAS signal variant. Because the sum of two minor allele frequencies of functional variants was close to the minor allele of the GWAS signal variant, there were three major halotypes (ABC, aBc and Abc) based on these three variants, so that the regressive model using two functional variants was able to explain the entire GWAS signal. This example was likely an unusual situation, and similar efforts have not been as successful.

Recent advances in biotechnology, including RNA sequencing and genome-wide chromatin immunoprecipitation (ChIP), have accelerated the identification of functional variants. It has been suggested that trait-associated variants are likely to be expression quantitative trait loci (eQTL; Nicolae *et al.*, 2010); however, there are difficulties in linking eQTL and disease variants due to tissue specificity (Heinzen *et al.*, 2008) and complex regulatory networks. More importantly, the problem that the most significant variant might not be the only functional variant remains, and controlling linkage disequilibrium (LD) is the only solution by which to solve these related issues. Variants associated with renal cancer have been found based on GWAS and genome-wide ChIP (Schodel *et al.*, 2012). Variants in the binding sites were in strong LD, constituting a haplotype; therefore, it was difficult to conclude which variants in the haplotype were responsible for the presentation of renal cancer (Schodel *et al.*, 2012).

Genome-wide functional studies could be useful for identifying direct associations (Ryu *et al.*, 2014), but it remains questionable whether the most significantly associated variant is the only true functional variant. Efforts have been made to identify disease variants in association studies using traditional approaches for confounders (Nicodemus *et al.*, 2004; Wrensch *et al.*, 2009) and advanced statistics for main associations (Charoen *et al.*, 2007; Szymczak *et al.*, 2009). These studies did not focus on identifying actual disease polymorphisms by controlling LD, which is the direct reason for the indirect association. More relevant studies have involved step-wise regressions for several associated variants in a locus (Cordell & Clayton, 2002; Biernacka *et al.*, 2007) and efforts have been made to identify polymorphisms that explain a linkage signal (Biernacka & Cordell, 2009) using family data. Step-wise regressions could be useful from a statistical point of view; however, by ignoring the actual relationship of LD, the method usually fails to identify actual disease variants when there is more than one disease variant in a locus (Park, 2010).

**Fig. 1.**
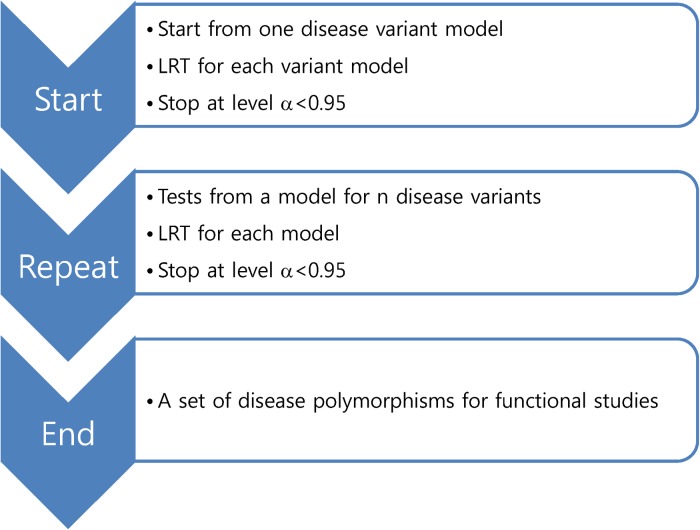
Procedure of identifying disease variants in case–control associations.

A method to address LD was previously developed (Park, 2007; Park, 2010). As shown in Fig. 1, the method employs an exhaustive procedure by testing all possible disease variant models. For an example with 10 variants in a gene locus, 10 likelihood ratio tests (LRTs) should be conducted for one-disease-variant models. Here, the distribution of LRT fits a chi-square with the given degree of freedom only for the correct model and not the remainder of the models. If one of the tests shows a p-value less than 0·95, then the variant model is the correct model. If none of the tests shows a p-value less than 0·95, 45 more LRTs should be conducted for two-disease-variant models. The LRTs continue until a model shows any p-values less than 0·95 when increasing the number of disease variants. As previously shown (Park, 2010), the method showed stably low Type I error rates and generally low Type II error rates. In Park, 2010, independent odds ratios of each variant were assumed; however, as shown in the allelic and locus heterogeneity of many Mendelian disorders (Nussbaum *et al.*, 2007), functional variants in a gene may show the same defective effects on gene function, leading to the causation of a complex disease. Therefore, it is necessary to study several disease variants in a gene having the same effects on the presentation of a complex trait. Additionally, the previous study was based on LRTs of alleles. Genotypic associations may be better for identifying actual disease variants to minimize possible influences of diploidy of the human genome.

## 2. Methods

The properties of disease variants were first examined according to models, to identify disease variants based on genotypic associations. The new method was then described, and simulation studies were provided to examine the validity of this method.

### (i) Disease model and disease variants with incomplete penetrance

**Fig. 2.**
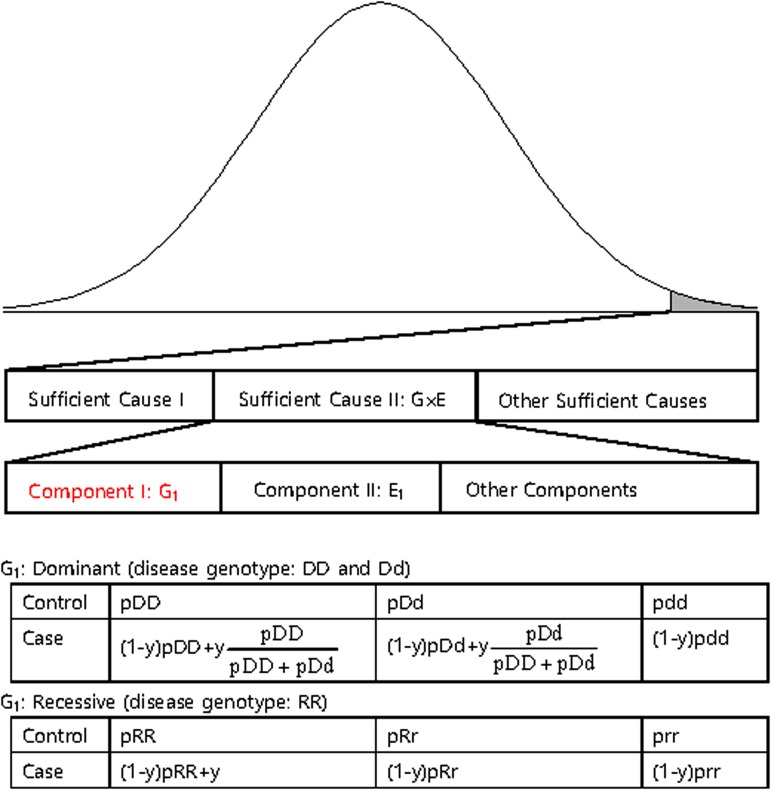
A sufficient causal component model and genotype frequencies of a genetic component of G × G or G × E.

The disease variants found from GWAS were neither sufficient nor necessary to cause disease because the discovered variants might not have been actual disease variants; otherwise, the interaction between the variant and other causal components, such as a disease variant in another gene or an environmental factor is also likely a compelling explanation. Recently, dissection of the causal factors of complex diseases was attempted based on the Sufficient Causal Component (SCC) model in epidemiology (Rothman *et al.*, 2008; Madsen *et al.*, 2011 *a*; Madsen *et al.*, 2011 *b*; Park & Kim, 2015). As shown in Fig. 2, a complex disease is presented in an individual when a SCC is fulfilled. SCCs could be single genetic factors, environmental factors, gene–gene interactions (G × G) or gene–environmental interactions (G × E).

Among SCCs, each causal component in G × G or G × E was not sufficient for presentation of the complex disease. Only when all the other causal components in G × G or G × E were fulfilled did an individual develop disease due to the SCC, G × G or G × E. Each causal component in G × G or G × E was not necessary for presentation of the disease because the disease could develop due to other causal factors. One of the causal components in G × G or G × E could be a gene with disease genotypes, and the behaviour of the disease variants in the gene was the same as those of variants discovered from GWAS, which were neither sufficient nor necessary for the presentation of complex diseases. In Fig. 2, an example of a genetic component in G × E is presented. The G_1_ component in Sufficient Cause II (G × E) could result in disease presentation only when all the other components in Sufficient Cause II were fulfilled. Additionally, an individual with a normal genotype of G_1_ can develop the disease due to Sufficient Cause I or other sufficient causes.

The G_1_ component could be dominant or recessive. If the population lifetime incidence (PLI) of the disease is small (approximately 1% of the population), as shown in Fig. 2, the genotype frequencies of controls are similar to those of the entire population. In the case population, the disease genotype frequencies would increase as a portion of y, which is the proportion of Sufficient Cause II in the PLI. In the case population, normal genotype frequencies would decrease as a portion of 1-y. There are two disease genotypes for a dominant gene; thus, each genotype proportion in the total disease genotypes should be considered, as shown in Fig. 2. Therefore, disease genotypes exist in the controls, and normal genotypes exist in the cases, as previously indicated.

**Fig. 3.**
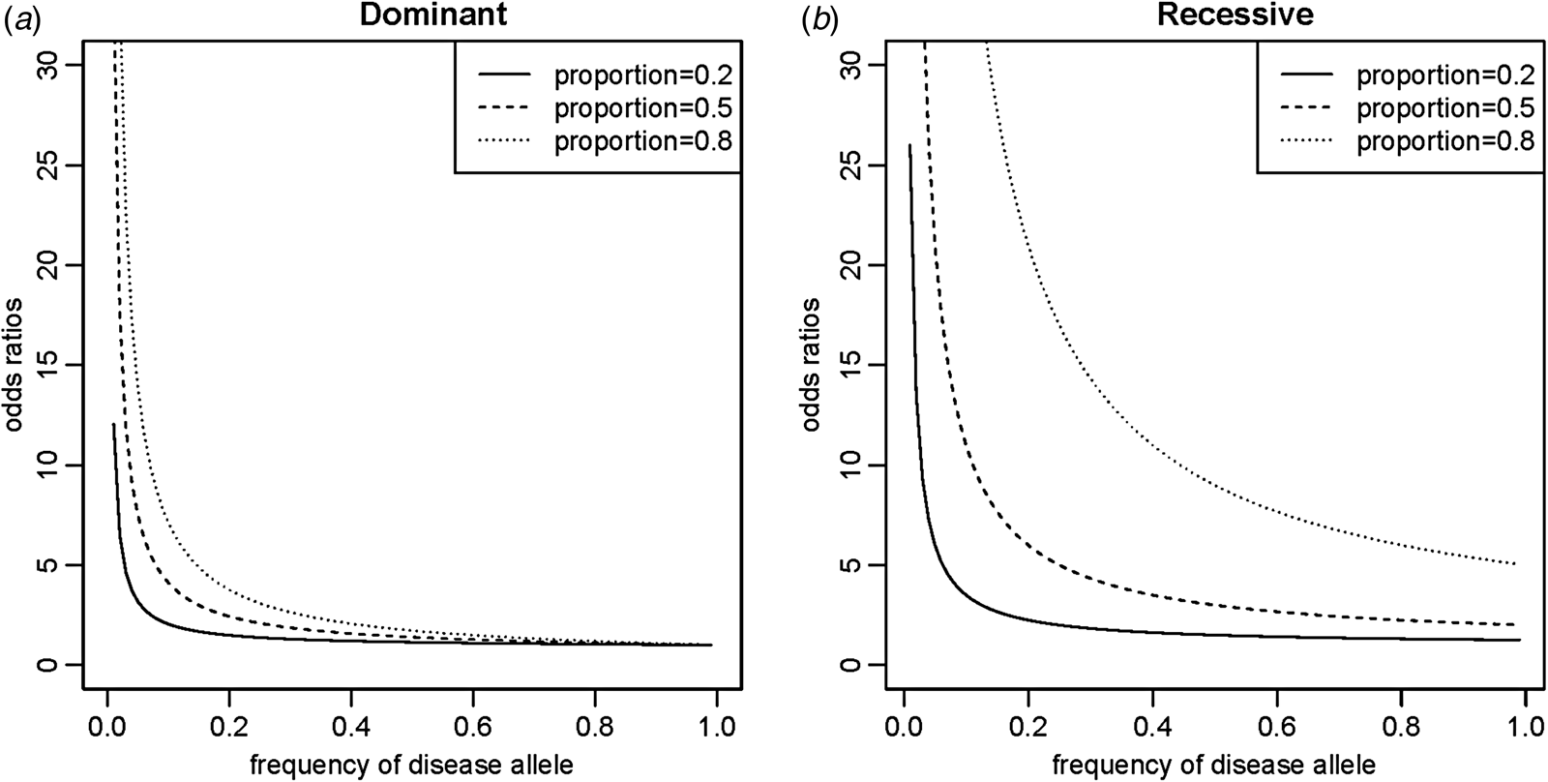
Changes in odds ratios depending on allele frequencies and proportions in population lifetime incidence. (*a*) Dominant genes; (*b*) recessive genes.

The odds ratios of the disease allele were different depending on their frequencies and proportions in PLI. As shown in Fig. 3, the odds ratios for a disease variant in a gene decreased as the frequencies of the disease allele increased for a fixed proportion, y, in PLI. The phenomena were more severe when the disease variant was dominant, showing odds ratios close to 1 for many variants with allele frequencies greater than 0·5. Considering the complexity of the causal components in the presentation of a complex disease, the proportions in PLI were small for the most sufficient causal components, and the odds ratios of common disease variants in a sufficient causal component were also small. For example, when the proportion was 0·2 and the allele frequency was 0·3, the odds ratio was 1·3, as shown in Supplementary Table 1. This result is reasonable considering that it is difficult for a severely defective allele to increase its frequency in a population due to purifying selection. For the full range of proportions, as the proportions become large, the odds ratios become extreme, especially when the disease variant is recessive, as shown in Supplementary Fig. 1. The penetrance of disease variants also decreases quickly as the proportions decrease and the disease allele frequencies increase. The genes associated with Mendelian diseases with incomplete penetrance and extreme odds ratios could be explained with this SCC model of G × G or G × E.

### (ii) Genotypic likelihood ratio tests based on SCC models

Unlike the previous study (Park, 2010), the current study assumed that disease variants in the same gene cause the same effects. Therefore, a haplotype was considered a disease haplotype if it contained more than one disease allele. Based on this property, it can be assumed that one virtual disease variant exists instead of all the disease variants in the gene. When considering genotypes, Hardy–Weinberg equilibrium (HWE) was typically assumed. However, in an actual situation, random sampling of gametes creates slight deviations from HWE depending on the population size and the sample size (Weir, 1996; Park, 2011). These deviations will be reflected in the sampled population as well. Therefore, in the current study, a method for identifying disease variants was developed based on genotypes with consideration of natural deviations from HWE.

The genotype frequencies of each variant in cases increased or decreased depending on the LD with genotype frequencies of the disease variant. To reflect the natural deviations from HWE, the current study employed the LD between genotypes in which the LDs between the alleles were reflected. By doing so, the population deviation from HWE could be correctly reflected in the analyses. The current study assumed that the disease variants in a gene gave the same effect on the disease presentation due to the malfunctions of the gene. If a haplotype contained two disease alleles, it would still be considered a disease haplotype. Therefore, regardless of how many disease variants exist in the gene, they can be considered to be only one disease variant in the gene. Each genotype frequency of a variant in cases could be expected as described below, depending on the LD with the genotype frequencies of the disease variant:
1
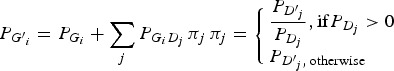


Here, P(G_i_´) indicates the ith genotype frequency in the cases, and P(G_i_) indicates the ith genotype frequency in the control. P(D_j_´) indicates the jth genotype frequency of the disease variant in the cases, and P(D_j_) indicates the jth genotype frequency of the disease variant in the control. P(G_i_D_j_) indicates the frequency in the control when an individual has both G_i_ and D_j_ genotypes. For D genotypes, there are only two alleles, disease and normal. Therefore, three genotypes are available, such as a regular bi-allelic variant.

To test whether a model of a disease variant is the true model, the likelihood ratio test was modified similarly to the previous study. In the current study, the number of possible genotypes was usually three for bi-allelic variants. Therefore, the likelihood would be based on the multinomial distribution instead of the binomial distribution. The variance corrections due to the control sampling from the actual population should be applied, similar to the previous study (Park, 2010). The likelihood ratio test for a variant can be expressed as follows:
2
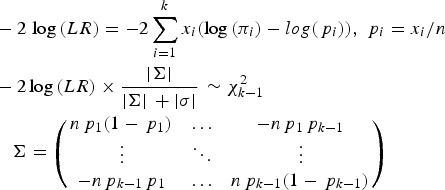


Here, k is the number of genotypes for the variant, and p_i_ is the genotype frequency for the ith genotype. π_i_ indicates the theoretical genotype frequency derived when the disease model is true. |∑| is the determinant of ∑, which is the variance and the covariance matrix for the k-1 genotypes. Because the frequency of the last element is completely dependent on all of the previous elements in multinomial distributions, ∑ includes up to (k-1)^2^ elements. Most variants are bi-allelic; therefore, ∑ is a 2 × 2 matrix in the current study. The degree of freedom for the k genotypes is k-1. The determinant of the simulated variance, |*σ*|, is a similar determinant to |∑|, which is approximately derived from random samplings to correct sampling errors for controls that are sampled from the actual control population. To generate the simulated variance, random samplings of the cases were performed 1000 times from the control population. The genotype frequencies of the disease variants in cases were first sampled based on the multinomial distributions with the genotype frequency probability of the target disease variant, and each individual case was reconstructed based on the genotype frequencies of a randomly sampled control individual with the corresponding genotype of the disease variant. For rare variants, controls may not have homozygous rare alleles, but cases may have such genotypes. To correct for such biases, 20% of the cases were sampled from those that had the same sampled genotype of the disease variant to obtain the simulated variance.

As shown in Fig. 1, the current method employed a similar procedure to identify actual disease polymorphisms. First, a model of one disease variant was tested for all the candidate variants. Each likelihood ratio test statistic was summed to examine the total likelihood, and the degree of freedom for testing n variants was 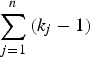. For bi-allelic rare variants that had only two genotypes, the degree of freedom was 1, which was k_j_-1. If several variants showed a p-value smaller than 0·95, the variant with the smallest p-value is the disease variant. If one of the genotypes showed a p-value smaller than 0·95, the variant was accepted as the disease variant in the gene. Otherwise, the next step was to continue to the model of two disease variants and test all the possible sets of variants. If one of the results showed a p-value smaller than 0·95, the set of variants was accepted as the disease variants in the gene. This newly developed method for computing the likelihood ratio test of the genotype frequencies was integrated into the existing R package (Identifying Functional Polymorphisms ‘IFP’: http://cran.r-project.org/web/packages/IFP/index.html).

### (iii) Simulations for estimating error rates

For the simulation data set, the sequencing data of the *APOE* region, which were known to be associated with Alzheimer's disease, from phase 1 of the 1000 Genomes Project were used (Abecasis *et al.*, 2010). The region including ± 1000 bp upstream and downstream regions of the gene was examined. The data consisted of 1092 individuals and 57 variants in the region. Among the 57 variants, 33 variants that had minor allele frequencies greater than 0·001 were included in the analyses. For each disease variant model, one virtual disease variant was derived in the control groups based on the actual disease variants. As shown in Fig. 2, the proportion (y) of PLI was derived from the odds ratio (OR) as follows:
3
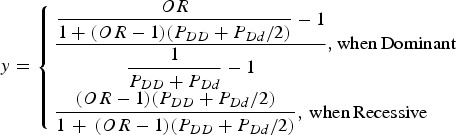


Here, P_DD_ and P_Dd_ are the genotype frequencies of the DD and Dd genotypes for the disease allele (D), respectively. When the variant was dominant, the proportion became larger than 1 for a certain odds ratio that was larger than 2(1-P_DD_)/P_Dd_-1. Therefore, certain high odds ratios are impossible for dominant variants with specific genotype frequencies.

Based on the proportion (y) and the genotype frequencies in controls, the genotype frequencies in the cases could be derived for a disease variant model. For specific sample sizes for the cases and controls, the control populations were directly sampled from the data of the 1000 Genomes Project. The number of disease genotypes in the cases were obtained by multinomial distribution with theoretical probabilities based on a dominant or recessive model, and the affected individuals were sampled from the same data based on the number of genotypes in the cases. When there was no homozygotes of rare alleles in the data of the 1000 Genomes Project, two haplotypes of the disease allele were randomly sampled. The sampled cases and controls were used to test which disease variant model was the true model. For most of the simulations, the sample sizes of the cases and controls were 500 unless otherwise specified, and 1000 simulations were conducted.

## 3. Results

### (i) One-disease-variant model

**Fig. 4.**
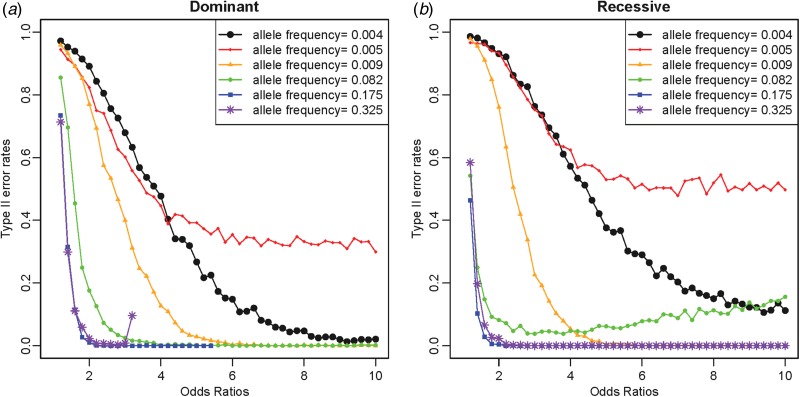
Type II error rates depending on allele frequencies and odds ratios. (*a*) Dominant variants; (*b*) recessive variants.

Similarly to the previous study (Park, 2010), Type I error rates were reasonably small at approximately 0·05 for the level *α* = 0·05 and approximately 0·01 for the level *α* = 0·01. The identification of the true disease variant model was based on all possible tests as shown in Fig. 1; thus, Type II error rates were also important, similar to the previous study (Park, 2010). Considering the importance of rare disease variants, the current study examined Type II error rates depending on various disease allele frequencies and odds ratios for the one-disease-variant model. As shown in Fig. 4, Type II error rates were higher overall for rarer disease variants; however, the LD patterns were more crucial for reducing Type II error rates. For rare disease variants with an allele frequency of 0·005, Type II error rates did not approach zero, even when the odds ratio was high. It was because the variant was in strong LD with another variant through r^2^ of 0·77. High Type II error rates were greater when the disease variant was recessive. The higher Type II error rates for recessive variants were observed for the other rare variants; however, the trend was opposite for common variants.

For common disease variants with allele frequencies greater than 0·05, Type II error rates were typically low for odds ratios greater than 2. For the dominant variant with a disease allele frequency of 0·325, Type II error rates increased for the maximum available odds ratios. The LD between the variants was examined in Supplementary Table 2, and it was clear that variant no. 18 with an allele frequency of 0·325 was in strong LD with variant no. 7, showing an r^2^ value of 0·446. The allele frequency of variant no. 7 was 0·510 greater than that of variant no. 18. For small odds ratios, the genotype frequencies of variant no. 7 were not sufficiently large even with the strong LD with variant no. 18; however, when the odds ratio was very large, the strong LD between these variants substantially increased the genotype frequencies of variant no. 7. Because variant no. 7 had greater frequencies, it was more likely to be detected as a disease variant than variant no. 18. Similarly, for the recessive variant with a disease allele frequency of 0·082, Type II error rates increased as the odds ratios increased. As shown in Supplementary Table 2, variant no. 15 was in strong LD with variant no. 23 through an r^2^ value of 0·502. Similarly, because the allele frequency of variant no. 15 (0·082) was smaller than that of variant no. 23 (0·149), the high odds ratios increased the genotype frequencies of variant no. 23. Therefore, variant no. 23 was identified as a disease variant ahead of the actual disease variant.

**Fig. 5.**
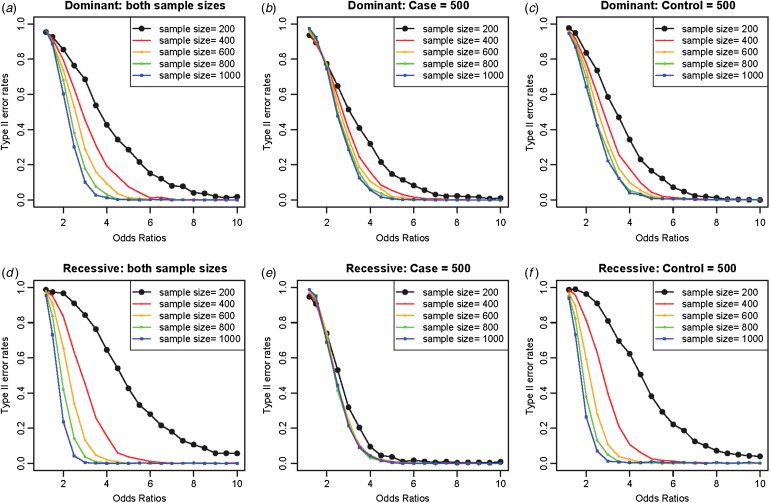
Type II error rates depending on sample sizes and odds ratios. (*a*) Dominant variants when both case and control sample sizes increase; (*b*) dominant variants when case sample size is fixed at 500 and control sample sizes increase; (*c*) dominant variants when control sample size is fixed at 500 and case sample sizes increase; (*d*) recessive variants when both case and control sample sizes increase; (*e*) recessive variants when case sample size is fixed at 500 and control sample sizes increase; (*f*) recessive variants when control sample size is fixed at 500 and case sample sizes increase.

Increasing the sample sizes was helpful to reduce the Type II error rates, as shown in Fig. 5, in which variant no. 10 with an allele frequency of 0·00870 was extensively examined as a disease variant. Different from the previous study (Park, 2010), which showed a larger effect of the control sample sizes in the reduction of Type II error rates, the current method showed a larger effect of the case sample sizes in the reduction of Type II error rates. The current study focused on rare variants, and variance corrections were partially based on the random sampling of the case samples. The control samples had relatively few disease genotypes compared with the case samples, especially for rare variants, which could lead to biased results depending on the sampled case individuals. Therefore, the larger effect of case sample size was more obvious when the disease variant was recessive, as shown in Fig. 5.

### (ii) Two-disease-variant model

**Fig. 6.**
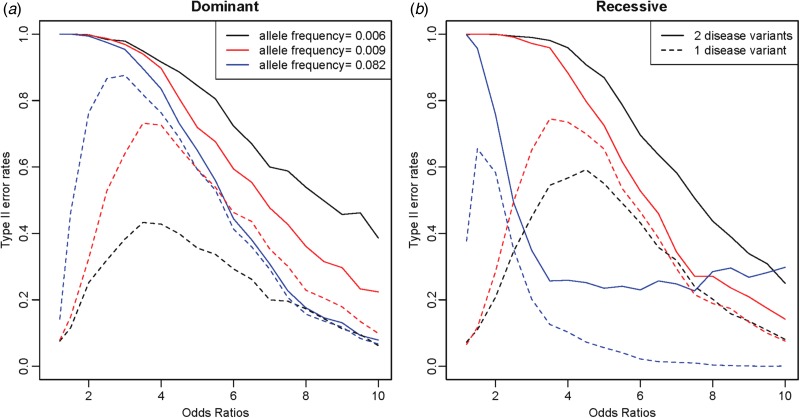
Two-disease-variant models for a fixed variant and a variant with various allele frequencies, in which the solid line indicates Type II error rates and the dashed line indicates the probability when only one of two disease variants is identified as a disease variant. (*a*) Dominant genes; (*b*) recessive genes.

When two disease variants exist in a gene region, Type II error rates can be increased because of the difference in allele frequencies between the variants. Different from the previous study based on haplotype associations (Park, 2010), in which the odds ratio of each variant was independent, the current study assumed the same influence of disease variants on disease presentation. The odds ratio of each disease variant was not independent; thus, a disease variant with larger frequencies could usually be identified as a disease variant during the procedure shown in Fig. 1 when the difference in allele frequencies between disease variants was large. Fig. 6 shows a simulation study of the two-disease-variant model. Variant no. 16 with an allele frequency of 0·00412 was fixed as the first disease variant, and the second disease variant varied depending on the allele frequencies as follows: variants no. 22, no. 10 and no. 15 had allele frequencies of 0·00592, 0·00870 and 0·0820, respectively.

In Fig. 6, the solid line indicates the Type II error rates when both disease variants were identified, and the dashed lines indicate the probability of the correct identification of only one of the disease variants. Overall, Type II error rates decreased as the disease allele frequency increased. As shown in Fig. 6(*a*), most Type II error rates came from the early identification of one of the disease variants when testing the one-disease-variant model. These phenomena were relatively less shown for disease variants with small differences between disease allele frequencies. When two disease variants had similar and small allele frequencies, the slightly high Type II error rates are shown as the odds ratios increased in Fig. 6. These results occurred because of the early identification of one of the disease variants and the incorrect identifications of other variants in LD with the disease variants, especially due to the strong LD (r^2^ = 0·768) between variants no. 22 and no. 2. For recessive variants, the frequency of only one genotype among three increased as the odds ratio increased. Therefore, the similar incremental effect of the genotypes of the other variants in LD could be more severe than that of the dominant variants, as shown in Fig 6(*b*) involving variant no. 15 in strong LD with high frequency variants.

**Fig. 7.**
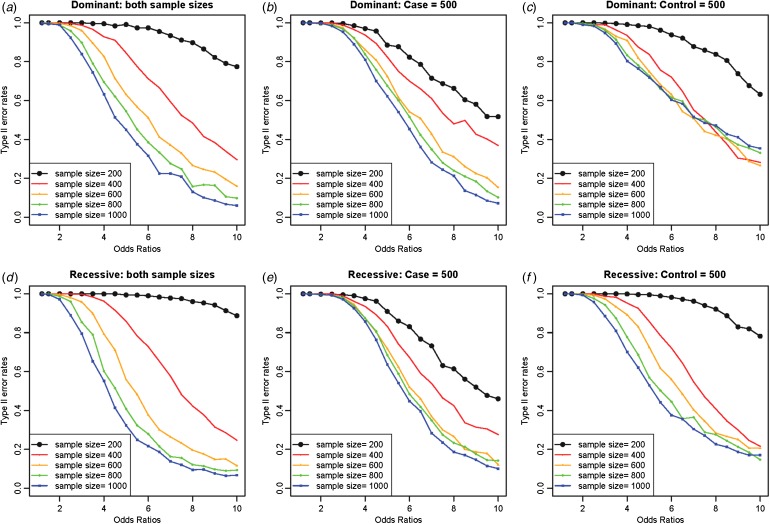
Type II error rates for two-disease-variant models depending on various sample sizes. (*a*) Dominant variants when both case and control sample sizes increase; (*b*) dominant variants when case sample size is fixed at 500 and control sample sizes increase; (*c*) dominant variants when control sample size is fixed at 500 and case sample sizes increase; (*d*) recessive variants when both case and control sample sizes increase; (*e*) recessive variants when case sample size is fixed at 500 and control sample sizes increase; (*f*) recessive variants when control sample size is fixed at 500 and case sample sizes increase.

As expected, increasing the sample sizes reduced the Type II error rates, as shown in Fig. 7. The plots were based on the model of two rare disease variants, variants no. 16 and no. 10, with allele frequencies of 0·00412 and 0·00870, respectively. Similar to Fig. 5, increasing the case sample size rapidly reduced Type II error rate; however, the Type II error rates did not decrease to zero as the odds ratios increased, especially for dominant variants with a fixed control sample size. The primary reasons for high Type II error rates were early identifications of more frequent variant and incorrect identifications of a variant with a larger allele frequency as the disease variant. Instead of identifying the rare disease variant, an incorrect common variant was detected as a disease variant due to strong LD through D´ with two disease variants, which coincidently increased its genotype frequencies in cases.

### (iii) Comparisons to the model of independent disease variants

Different from the previous study (Park, 2010), increasing the case sample sizes was slightly more effective in decreasing Type II error rates. The previous study targeted common disease variants so that the allele frequencies and LD relationships of the control populations were important for correct identification. However, in the current study, rare variants were of major interest and variance corrections used both the control and case samples, so the genotype frequency constitutions were not dependent only on the control samples. For recessive variants, increasing the case sample sizes was slightly better than increasing the control sample sizes. Different from dominant variants, only one genotype frequency increased in cases for recessive variants. In addition to the rare disease genotype frequencies in controls for rare variants, the genotype frequency of heterozygotes of the disease variant was rare in the controls and slightly rarer in the cases of recessive variants according to Fig. 2. Therefore, increasing sample sizes reduced Type II error rates more substantially for recessive variants than for dominant variants.

Similar to the previous study (Park, 2010), the method provided good performance for identifying common disease variants with allele frequencies greater than 0·05, as shown in Supplementary Fig. 2, except in the condition in which two disease variants were in strong LD. Two common disease variants, no. 15 and no. 33 (red lines in Supplementary Figs 2(*c*) and 2(*d*)), were in strong LD with an r^2^ value of 0·410 (Supplementary Table 2). In this case, the increments of disease genotype frequencies in the cases were always dependent on the frequent variant (no. 33); the frequent variant was identified as the disease variant when testing a model of one disease variant. The phenomena were more significant than those in the previous study (Park, 2010) because the effects of the two disease variants were independent in the previous study. To discriminate the effect of these two disease variants with the same effect on disease presentation, more studies are necessary.

## 4. Discussion

The current study presents an alternative method for identifying disease variants based on genotype frequencies when the disease variants have the same effects on disease presentation. The method works best for common variants with high impacts; however, with large sample sizes, it works reasonably well for rare variants with high impacts, especially variants that are in high LD through D´ rather than through r^2^, which means that the rare allele of each variant is associated with the common allele of another variant. These situations of LD are expected to be observed frequently because most rare variants are in high LD through D´ but not through r^2^. The previous method is suitable for the dense genotyping of a locus harbouring several independent disease variants (Park, 2010), and the current method is suitable for re-sequencing data harbouring several disease variants with the same impact on disease presentation. With more developments on disease variants in strong LD through r^2^, the current method may provide an ultimate solution for identifying true disease variants in conjunction with the previous method by identifying both dependent and independent disease variants.

However, the method needs to be improved to reduce the Type II error rates for rare disease variants. The correct identification of all disease variants is difficult, especially when two or more disease variants are in high LD through r^2^. Therefore, even though a disease variant was identified through the current method, caution should be taken regarding the possible existence of another disease variant in high LD through r^2^. Possible solutions to identify all of the disease variants might be the observations of Hardy–Weinberg disequilibrium for disease variants (Lee, 2003; Song & Elston, 2006; Grover *et al.*, 2010; Gao *et al.*, 2012; Xu *et al.*, 2012) or analyses of the variants of the gene locus that interact with the analyzed gene locus (Phillips, 2008; Cordell, 2009; Shin *et al.*, 2012).

The current study provides reasonable explanations regarding the low odds ratios of common disease variants and the high odds ratios of rare disease variants based on new genetic models (Park & Kim, 2015). Additionally, the study also clearly explains why disease variants of common complex diseases are neither necessary nor sufficient for the disease presentation based on the G × G or G × E interactions. Because the current method assumed that the control sample represented the entire population for making the simulation data set, the usage of cohort populations regardless of disease status works best for the assumption rather than the regular control samples. However, the result would not differ unless the PLI of complex diseases and the proportion of the corresponding causal component were high. It is also noteworthy that the method is independent of the disease model because the LRT depends entirely on the changes of disease genotype frequencies and on the LD between disease variants and other variants.

This study assumed a G × G as a complementary interaction, in which all of the interacting genes should have disease genotypes for a disease phenotype. In a previous study (Park & Kim, 2015), the complementary gene interactions and epistasis came from existing genetic observations; however, in fact, it cannot be ruled out that other types of complicated gene interactions might also exist. In this case, alternative explanations for the changes in odds ratios depending on allele frequencies would be required, and the simulation data should be rebuilt. Even in this case, the proposed method still could identify disease variants because the method is independent of disease models as indicated previously. In addition, since it is convenient to reduce the possibilities to two (function or malfunction), the assumptions still might offer advantages.

## Supplementary material

For supplementary material accompanying this paper visit https://doi.org/10.1017/S0016672316000033. click here to view supplementary material
