# Supplementary material for: Rare high-impact disease variants: properties and identifications
Source: Genet Res (Camb). 2016 Mar 21;98:e6. doi: 10.1017/S0016672316000033 (PMC6865157; doi:10.1017/S0016672316000033)
Supplement: Supplementary file 1 [file S0016672316000033sup.zip › S0016672316000033sup001.docx]

Supplementary Materials

Supplementary Fig. 1. Odds ratios and penetrance depending on disease allele frequencies and the proportion of the corresponding causal component in PLI: A. Odds ratios for dominant variants; B. Odds ratios for recessive variants; C. Penetrance for dominant variants; D. Penetrance for recessive variants.

Supplementary Fig. 2. Two-disease-variant models for a fixed variant and a variant with various allele frequencies, in which the solid line indicates Type II error rates and the dashed line indicates the probability when only one of two disease variants is identified as a disease variant: A. Dominant genes for a fixed variant with a disease allele frequency of 0.009; B. Recessive genes for a fixed variant with a disease allele frequency of 0.009; C. Dominant genes for a fixed variant with a disease allele frequency of 0.082; D. Recessive genes for a fixed variant with a disease allele frequency of 0.082.

Supplementary Table 1. Odds ratios and penetrance depending on disease allele frequencies and the proportion of the corresponding causal component in PLI: A. Odds ratios for dominant variants; B. Odds ratios for recessive variants; C. Penetrance for dominant variants; D. Penetrance for recessive variants.

Supplementary Table 2. LD between variants in *APOE* from the 1000 Genomes Project.
