# Supplementary figures and images for: Rare high-impact disease variants: properties and identifications
Source: Genet Res (Camb). 2016 Mar 21;98:e6. doi: 10.1017/S0016672316000033 (PMC6865157; doi:10.1017/S0016672316000033)

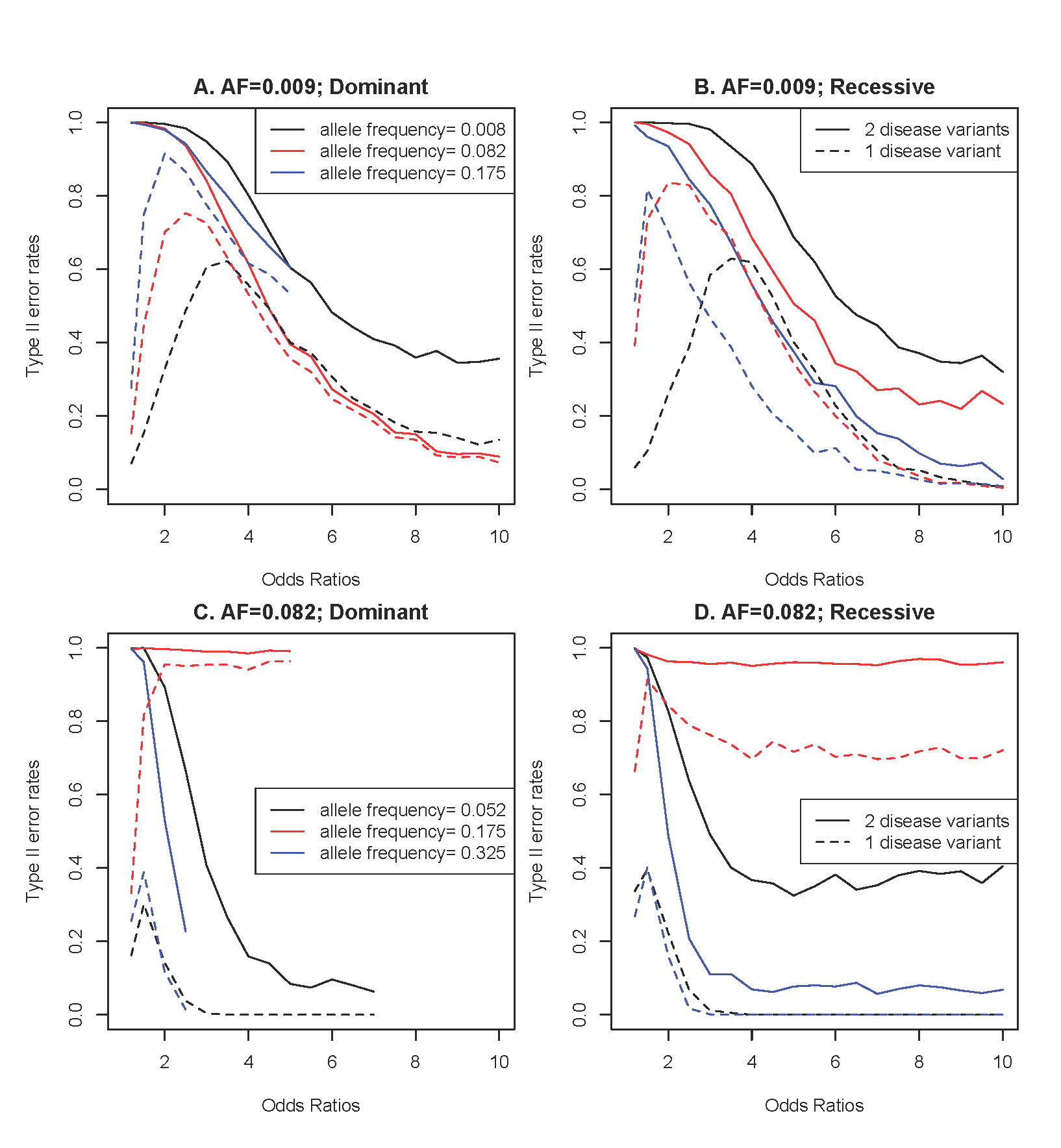

Supplement: Supplementary file 1 [file S0016672316000033sup.zip › S0016672316000033sup003.tiff]
